# A neurobiology perspective on the assembly of retinal vasculature from 2D to 3D

**DOI:** 10.1016/j.conb.2025.103085

**Published:** 2025-07-15

**Authors:** Mahima Bose, Mengya Zhao, Kenichi Toma, Xin Ye, Xin Duan

**Affiliations:** 1 Department of Ophthalmology, School of Medicine, University of California San Francisco, San Francisco, CA, USA; 2 Institute for Integrated Cell-Material Sciences (iCeMS), Institute for Advanced Study, Kyoto University, Kyoto, Japan; 3 Department of Discovery Oncology, Genentech Inc., South San Francisco, CA, USA; 4 Department of Physiology, Kavli Institute for Fundamental Neuroscience, University of California San Francisco, San Francisco, CA, USA

## Abstract

The reciprocal regulation of the neural ensemble and vascular network within the mammalian central nervous system (CNS) is crucial for its development and functionality. Neuron-derived pro-angiogenic factors, such as growth factors, morphogens, and guidance cues, play a key role in forming stereotypical vascular architectures in the cortex, spinal cord, and cerebellum during development. Notably, the CNS vasculature forms distinct 3D lattice structures composed of laminar vascular networks interconnected by penetrating vessels. This contrasts with the more random 3D arborizations found in tumors. While the morphogen gradients for vascular network growth have been well-studied, the mechanisms contributing to vascular patterning and lattice maintenance in 3D are not fully understood. The mammalian retina provides an ideal model for studying these mechanisms, given its laminar organization of neurons and plexus organization of vessels, allowing for the investigation of 2D growth to 3D lattice establishment in a stepwise manner. Notably, recent studies have highlighted the roles of neurons and glia in retinal vascular patterning in 2D, as well as the involvement of neurotransmitters in regulating vascular growth. Additionally, direct neuron-to-vessel interactions have been found to contribute to 3D retinal vascular lattice formation. As emerging technologies provide new insights into retinal vascular assembly in 3D, understanding the developmental regulation and the physiological and pathophysiological effects of 3D lattice disruption remains a fertile field of research.

## Introduction

The assembly of neural circuits is coordinated with the establishment of the vascular network throughout the central nervous system (CNS). The intricate arrangement across the neural and vascular systems ensures sufficient oxygen and nutrient supply from the vessels onto the neurons [1,2]. Multiple growth factors, morphogens, and guidance cues are shared across the neural and vascular systems, intertwining their developmental timelines [3,4]. Early mechanistic insights into how neurons affect blood vessels emerged from studies of developing peripheral nerves [5,6]. These studies demonstrated that peripheral nerves play a critical role during embryonic development by guiding blood vessel branching and arterial differentiation in the skin. In the mouse CNS, during embryonic development [7–9], specific neuron-derived angiogenic signals can be identified by examining cross-sections of the spinal cord or brain, showing a temporal and spatial restricted manner during development in a two-dimensional (2D) format.

Key morphogens responsible for the formation of two-dimensional vascular networks have been identified through traditional genetic and developmental biology approaches. However, the vascular network forms a more complex three-dimensional (3D) lattice structure composed of layered vascular networks connected by penetrating vessels. Such an arrangement is essential for supporting volumetric neural functions in 3D. The CNS vasculature is distinct due to its 3D lattice-based organization, setting it apart from the random arborization found in other systems, such as tumors. Yet, the mechanisms that contribute to the transition from 2D radial migration to 3D lattice organization remain unclear. Moreover, the physiological and pathophysiological consequences of the disruption of this 3D vascular organization remain to be determined.

**Figure 1:**
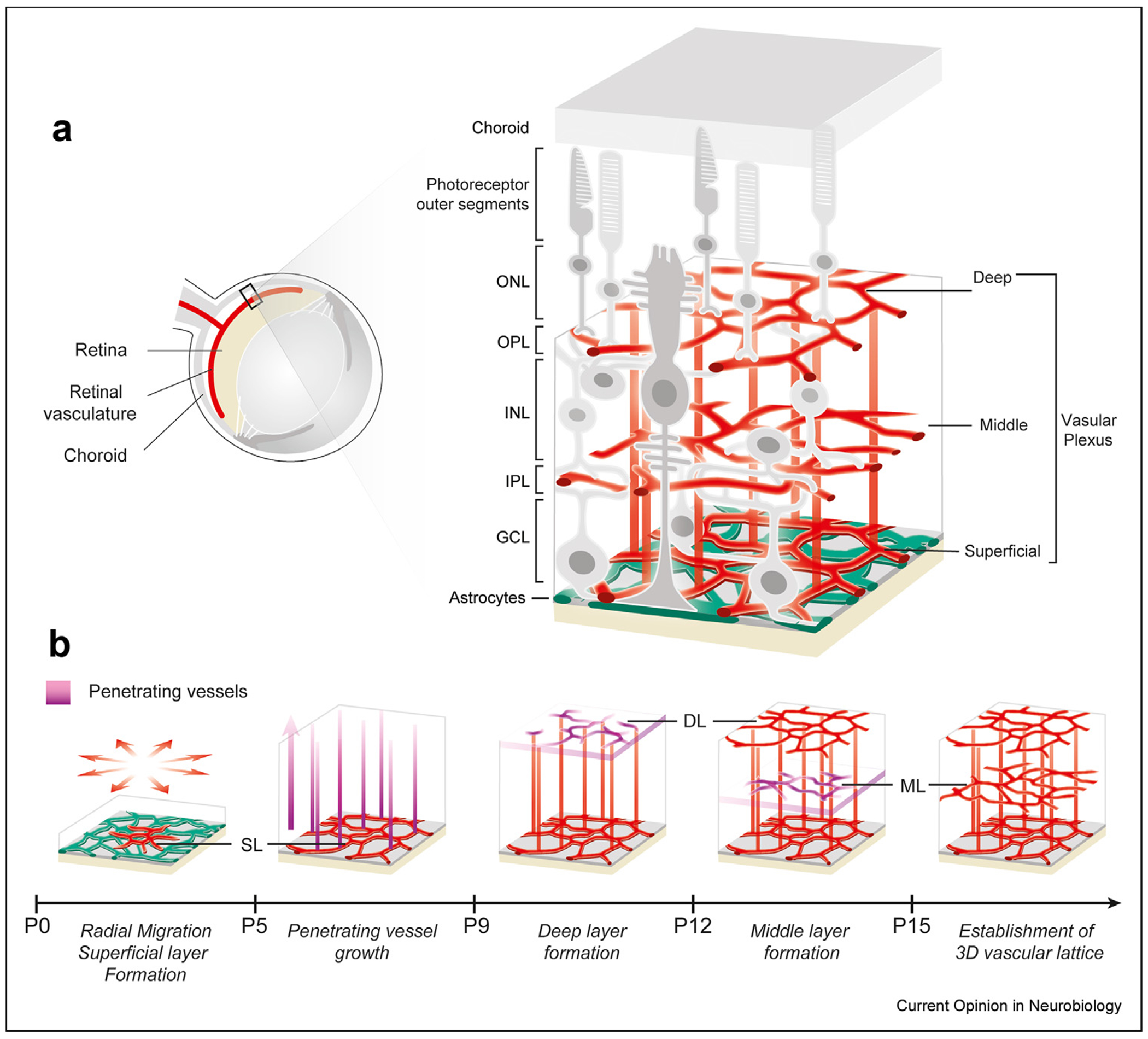
Development of retinal vasculature: synchronized neural and vascular growths. **(a)** Retinal cross-section illustrates the neuronal layers alongside their associated vascular plexuses. **(b)** Developmental timeline of retinal vasculature during early postnatal stages. Endothelial cells (ECs) migrate on the astrocytic network at P0, forming the Superficial Layer (SL). The ECs then invade the retinal tissue P5 onwards, creating the Deep Layer (DL) first, followed by the Middle Layer (ML), culminating in the characteristic 3D lattice structure of the retinal vasculature. Additional abbreviations: ONL, Outer Nuclear Layer; INL, Inner Nuclear Layer; GCL, Ganglion Cell Layer; OPL, Outer Plexiform Layer; IPL, Inner Plexiform Layer.

The retina is one of the most widely used models for studying angiogenesis, vascular patterning, and vascular dysfunction [10,11]. In mice, retinal vascular patterning undergoes stepwise development from 2D to 3D postnatally over weeks (Figure 1). The retina’s distinct neuronal layers and laminar vasculature provide a clear system to explore how neurons regulate the organization of the 3D vascular lattice. Beyond the conventional molecular and genetic knowledge, recent single-cell transcriptomic approaches have helped provide molecular definitions for each neuronal type [12]. This single-cell atlas has significantly advanced the identification of molecular markers for specific neurons. Furthermore, the mouse retina is an easily accessible system for imaging-based approaches, which allow a precise visualization of vascular changes within the architecture of the eye [13,14]. Re-engineered adeno-associated viruses (AAVs) optimized for transvascular neuronal labeling enable spatial mapping of neurons relative to nearby vessels based on the differential trans-vascular abilities of AAV serotypes [15].

In adults, the regulation of retinal blood flow is controlled by the neurovascular unit (NVU). This unit is made up of different types of cells, including neurons, vascular cells (such as endothelial cells, pericytes, and smooth muscle cells), and glia (including Müller glia, astrocytes, and microglia). These cells together form the structural basis of the Blood-Retina-Barrier (BRB). This coordinated structural composition allows for the selective dilation and contraction of blood vessels to meet metabolic demands. This process, known as neurovascular coupling, is crucial for the function of the NVU. Reviews on BRB formation and function [16–18], as well as the regulation of the adult NVU [19–23] cover these topics, which are beyond the scope of the current review.

Here, we focus on the development of retinal blood vessels and expand on recent findings that highlight the roles of neurons and glial cells in guiding the 2D patterning of retinal blood vessels, with particular emphasis on the underlying cellular and molecular mechanisms. We discuss how neuronal activity and the release of neurotransmitters from retinal neural cells influence the growth of blood vessels from 2D to 3D growth. Additionally, we highlight recent progress in understanding how interactions between retinal neurons and blood vessels contribute to the 3D organization of the vascular network. Finally, we explore the potential physiological and pathological consequences of disruptions in this 3D vascular network and discuss new technologies that are providing fresh insights into neurovascular mechanisms.

### A stepwise assembly of retinal vasculature from 2D to 3D

The embryonic retina is nourished by two extra-retinal vascular systems: the choroidal vasculature, which supplies the outer retina, and the hyaloid arteries, which nourish the inner retina and lens. While the choroidal vasculature persists throughout life, the hyaloid arteries are transient and are replaced postnatally by a dedicated intraretinal vascular system [24]. The development of the intraretinal vascular system is initiated as endothelial cells (ECs) migrate into the retina from the optic nerve head. These endothelial cells undergo proliferation and radial migration along the 2D surface of the retina till the vascular plexus reaches the periphery. During this stereotypical 2D growth phase, ECs differentiate into major arteries, veins, and capillary networks (Figure 1b). In mice, this developmental process occurs within the first postnatal week.

Beginning around postnatal day (P) 7–8, retinal vascularization in 3D involves ECs developing into three planar vascular beds connected by vertically penetrating vessels, which form sequentially. The first layer, the superficial layer (SL), forms near the vitreal surface through the initial radial migration of ECs. This is followed by the formation of pillar-like vertical sprouts that penetrate through the retinal ganglion cell layer (GCL) into the inner retina. These vertical sprouts lead to the formation of a second capillary bed, known as the deep layer (DL). After the DL is established, a new set of vertical sprouts emerges from the DL, producing the middle layer (ML) between the SL and DL, thus completing the formation of the 3D retinal vascular lattice (Figure 1).

In mice, this entire retinal vascular growth primarily occurs during the first 3–4 weeks after birth, coinciding with the formation and refinement of neural circuits [25,26]. Throughout this process, multiple cell types—including RGCs, Amacrine Cells (ACs), Müller glia, and brain-derived astrocytes—play essential roles in dynamic cell–cell interactions that guide retinal vascularization [8,27]. These interactions are mediated via multiple spatiotemporally regulated signaling pathways and contact-based mechanisms, ensuring proper vascular morphogenesis and BRB formation.

### Pro-angiogenic growth factors and morphogens in 2D patterning

**Figure 2:**
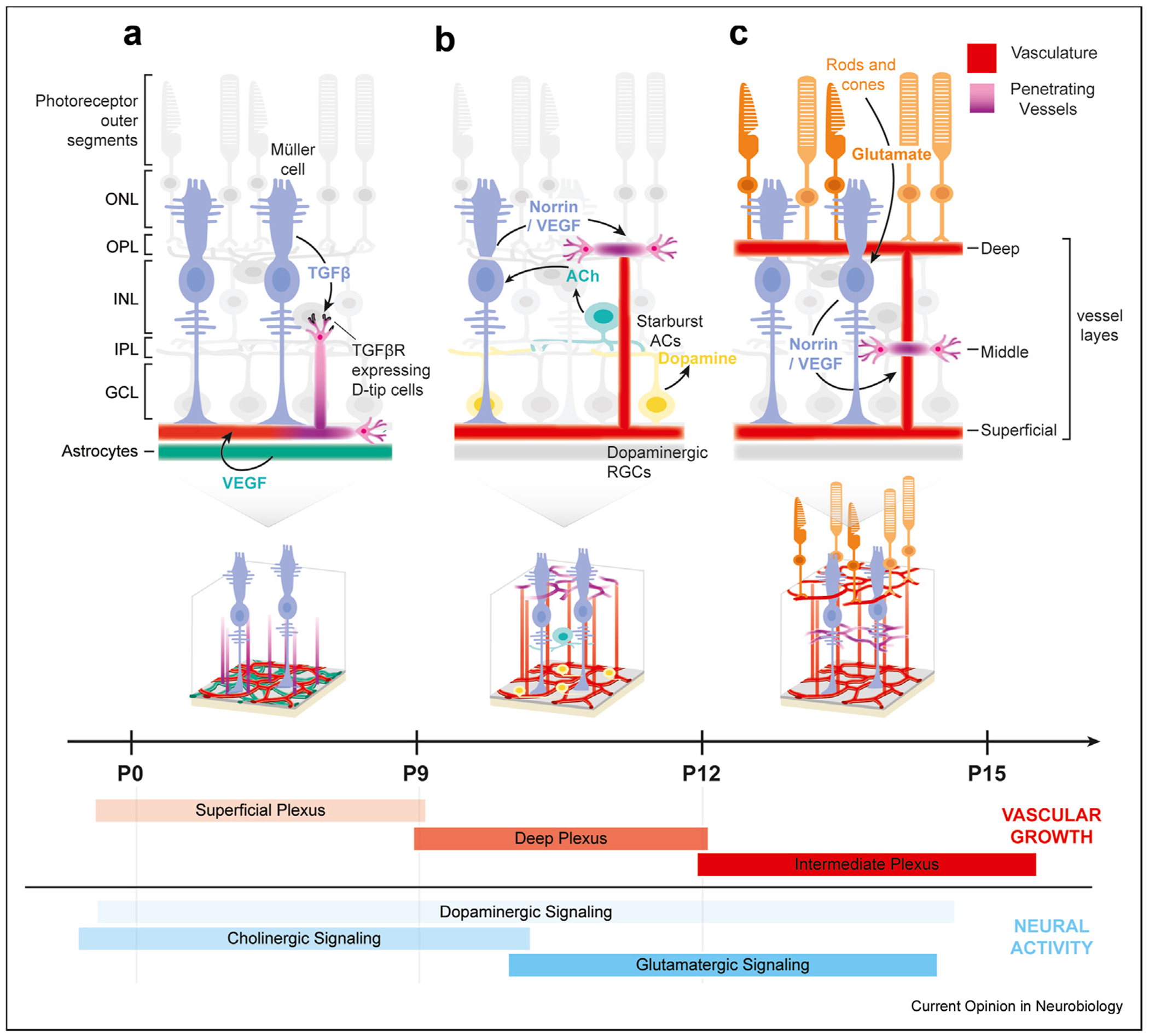
Diffusible factors and neurotransmitters from neural retina regulating retinal vasculature. **(a)** VEGF promotes endothelial cell (EC) migration and initial Superficial Layer (SL) vascular growth from astrocytes and Müller glia. Initial EC invasion into retinal tissue is mediated by TGFβ signaling from Müller glia onto D-Tip ECs, expressing TGFβ receptors. **(b)** Müller glia continues releasing pro-angiogenic factors, including Norrin and VEGF, onto ECs. Starburst amacrine cells (SACs) release acetylcholine (Ach), while dopaminergic retinal ganglion cells (RGCs) and amacrine cells (ACs) release dopamine, further enhancing Müller glia-driven angiogenesis. **(c)** Neural activity-dependent glutamate release, mainly from photoreceptors, amplifies Müller glia-driven angiogenesis via the Norrin pathway. Correlated vascular plexus growth is labeled in red; distinct neurotransmitters are labeled in blue along the first two weeks of retinal development.

One of the primary inducers of angiogenesis is tissue-hypoxia-induced Vascular Endothelial Growth Factor (VEGF) signaling. Foundational studies by Stone et al. [28] and others demonstrated that VEGF is transiently expressed in multiple retinal cell types, and its temporal expression precedes the ingression of ECs. The characterization of VEGF as a highly critical factor in angiogenesis was initially motivated by case studies on retinopathy in premature infants, where excessive oxygen exposure suppressed retinal vascularization [29–31]. Retinal astrocytes serve as a major source of VEGF during the initial 2D vascular expansion. Migrating from the brain during early development, these astrocytes associate closely with RGC axons, forming a sub-strate for EC migration (Figure 2a) [8,27]. RGC axons provide directional cues to the astrocytes, which in turn secrete VEGF to promote angiogenesis. Following astrocytes, Müller glia in the inner nuclear layer (INL) also contribute to VEGF signaling later in development [30]. This VEGF expression guides EC invasion into the retina (Figure 2a) [32]. In addition to glial cell-derived VEGF, RGCs also express VEGF during the development period. While the absence of VEGF from astrocytes or Müller glia does not entirely inhibit angiogenesis, the loss of RGCs halts angiogenesis even in the presence of astrocytes [33–35], highlighting a key role of neuron-derived VEGF in promoting vascular growth. This VEGF expression in RGCs is regulated by the succinate receptor GPR91 and the coagulation factor II receptorlike 1 (*F2rl1*) [34,36]. Additionally, VEGF receptors (VEGFR2) expressed by RGCs and RGC subtypes can titrate the amount of VEGF in the environment to regulate retinal vascularization [37]. Recent studies also suggested that ACs and Horizontal Cells also express VEGF in an HIF1α-dependent manner, contributing to the sprouting of vessels in the deep and middle vascular plexuses [31]. These studies confirm the crucial role of VEGF in vascular growth, orchestrated in a spatiotemporally regulated manner (Figure 2a).

In parallel, the TGFβ signaling pathway has also been implicated in the neural regulation of angiogenesis in the retina. Earlier work showed that the deletion of the TGFβ Type-II receptor (*Tgfbr2*) in ECs prevents the formation of the vascular plexus at the DL [38]. A recent study, enabled by single-cell RNA-sequencing technology to characterize EC diversity, revealed the molecular differences between endothelial tip cells driven by TGFβ signaling. A subtype of ECs, called S-Tip cells, vascularizes the SL, while another type, called the D-Tip cells, targets the DL (Figure 2a). D-Tip cells, distinguished by the expression of TGFβ Receptor (*Alk5*), rely on this gene for proper fate specification. Loss of this receptor from the D-Tip cells disrupts DL vasculature and alters pericyte function. The TGFβ ligands, responsible for guiding this vascular organization, were shown to be expressed by the Müller glial cells [39]. This study introduced novel and distinct EC subtypes into the landscape of retinal vascular development. Understanding the differential properties of these EC subtypes in their responses and interactions with neighboring cells is crucial for elucidating the mechanisms of vascular development [39].

Another prominent example of a secreted factor in retinal vascular growth is Norrin’s role in the inner retina. Norrin is a distant member of the TGFβ superfamily, with structural similarities to TGFβ but without any known interactions with the TGFβ receptors. It is highly expressed in Müller glia [40]. Norrin plays a key role in activating Wnt signaling in ECs as a non-Wnt ligand [41]. Norrin binds to the Frizzled receptor (*Fzd4*), the co-receptor *Lrp5*, and the co-activator *Tspan12* to regulate both angiogenesis and the integrity of the blood-retina barrier (Figure 2b) [42–44]. The restricted action of Norrin and downstream *Lrp5*/*Tspan12* made them druggable targets to activate ECs for vascular restoration in the adult retina in disease settings [45].

Furthermore, multiple short-range guidance cues from neurons also induce angiogenesis, which were systematically reviewed here [46,47]. Specifically, in the retina, RGC-derived Sema3E signals through PlexinD1 in ECs to regulate retinal vascular development [48,49]. The absence of either Sema3E or PlexinD1 resulted in a disrupted vascular front and an overall simpler vascular network [49]. Phenotypes of known mouse mutants impacting retinal vasculature are also summarized in Table 1 to reflect recent progress beyond those summarized previously [8,9,46].

### Retinal neurons communicating with the vasculature

Neuronal activity is widely regarded as a critical factor in promoting vascularization, including both 2D radial growth and 3D arborization. Multiple lines of evidence suggest that sensory deprivation reduces EC proliferation and vascular density in the retina [50]. For instance, retinal vascular development depends on the Melanopsin-driven light-responsive pathway. This discovery emerged from a well-designed genetic strategy to identify one of the most well-characterized RGC types across species: the intrinsic-photosensitive RGCs (ipRGCs) [51–53]. Studies show that genetic ablation of the Melanopsin gene (*Opn4*) in mice or dark rearing of rodents impairs VEGF expression, which prevents the regression of the hyaloid vasculature by P8 and leads to an abnormal retinal vascularization [54]. However, the precise mechanistic link between the loss of *Opn4* and VEGF impairment remains to be determined, especially in terms of the specific neuronal roles of *Opn4*-positive ipRGCs in impacting vasculature during the embryonic stages. A recent study identified another non-overlapping melanopsin-expressing cell type, Opn5^+^ RGCs [55]. During the early first postnatal week, stimulation of Opn5^+^ RGCs by 380nm violet light delays hyaloid regression by increasing the expression of the dopamine reuptake transporter (DAT) in the inner retina, thereby modulating dopamine availability. This dopaminergic signaling cascade contributes to the gradual regression of the hyaloid vessels by downregulating VEGFR2 expression in hyaloid ECs, ultimately reducing their survival.

Second, focusing on other retinal neuron types, one of the best-characterized mouse retinal interneuron types is the starburst amacrine cells (SACs), which provide cholinergic inputs to the inner retina [56]. Blocking SAC activity or reducing SAC numbers leads to an impaired EC invasion and BRB leakiness. The cholinergic neuronal inputs from these neurons modulate VEGF signaling for regulating angiogenesis and independently modulate the Norrin/Fzd4 signaling pathway for maintaining BRB function [57] (Figure 2b).

Third, expanding the conventional neurotransmitter pool, various genetic and functional evidence demonstrates that excitatory glutamatergic neuronal activity also promotes angiogenesis, particularly in the DL, and supports the maturation of the BRB. Specifically, active glutamate release, mainly from excitatory retinal neurons, boosts Norrin expression from Müller glia and interneurons to promote angiogenesis [58] (Figure 2c).

Fourth, RGCs also release dopamine during early post-natal development, which negatively regulates the growth of the vasculature during its active expansion. Specifically, dopamine release from RGCs inhibits the Jagged-Notch pathway, serving as an inhibitory mechanism downstream of the interaction between VEGF and VEGFR to regulate early blood vessel formation in the SL. This inhibition reduces the sprouting of tip cells and limits the expansion of the vascular network [59] (Figure 2b). In contrast, dopaminergic ACs, the main source of dopamine in adults, do not play a role in vascular growth, likely due to its late onset of dopamine release.

### Retinal neurons and non-neurons directly interact with vessels for 3D vascularization

**Figure 3:**
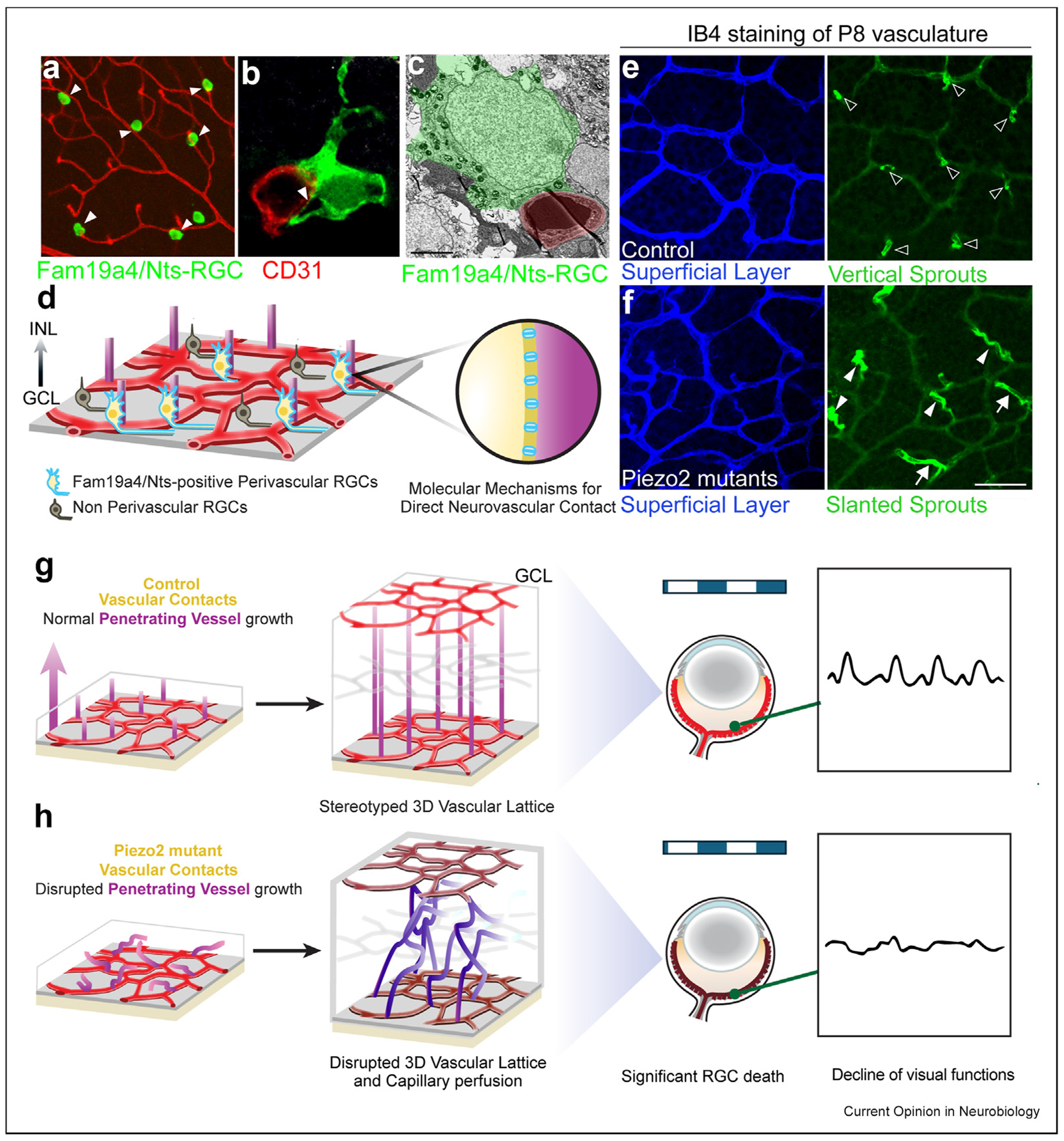
Perivascular RGCs pattern 3D Vascular lattice formation and impact retinal vascular and neural functions. **(a–c)** Direct interactions between perivascular RGCs (Fam19a4/Nts-RGCs) and nearby penetrating vessels (arrowheads) guide 3D retinal vascular patterning. **(d)** Fam19a4/Nts-RGCs, as perivascular neurons, mediate direct neuron–vascular interaction via PIEZO2-dependent (**d**’: in blue) mechanisms. (**e–f)** Comparative IB4 staining of retinal vasculature shows that neuronal *Piezo2* conditional mutants in (F; filled arrowheads) disrupt penetrating vessel growth compared to control (**e**; hollow arrowheads). **(g–h)** The same *Piezo2* mutants **(h)** develop into abnormal 3D vascular structures in adults, leading to functional consequences, including impaired capillary perfusion, substantial RGC loss, and vision deficits. Scale bars: **(a)**: 60 μm; **(b)**: 5 μm; **(c)**: 3 μm; **(e, f)**: 50 μm (**a-c**, **e** &**f** were adapted from Toma et al., 2024, *Cell*).

The examples discussed above mainly focus on morphogens, growth factors, guidance cues, and neuro-transmitters in shaping the growth of blood vessels. However, the proximity between neurons and the vascular scaffold also suggests that specific retinal neurons or groups of neurons could preferentially localize to the perivascular space and affect the vascular network, including their 3D arrangement, through direct physical interactions. A recent viral labeling and genetic study identified a specific subtype of RGCs, termed Fam19a4/Nts-RGCs, as perivascular neurons involved in controlling the 3D vascular lattice formation via direct contacts [60]. Fam19a4/Nts-RGCs are primarily located in the perivascular space, anchoring onto the scaffold of the penetrating vessels. These RGCs serve a critical role in supporting the proper orientation of penetrating vessels, ultimately regulating the 3D vascular lattice organization (Figure 3a, b, c). At the molecular level, this neurovascular interaction is mediated by the mechano-sensitive ion channel PIEZO2, which was found to be enriched in these RGCs (Figure 3d). Genetic perturbation experiments revealed that the absence of *Piezo2* from either *Nts*-positive perivascular RGCs or all RGCs led to a disrupted 3D vascular lattice, resulting in a phenotype not previously reported in any other mouse mutants (Figure 3e and f). The disruption of the vascular lattice structure due to *Piezo2* loss in these neurons leads to long-lasting functional consequences, such as reduced vascular perfusion rates, chronic hypoxia, progressive RGC loss in the mature retina, and enhanced susceptibility to ischemic ocular insults (Figure 3g and h). This finding is significant in the context of retinal angiogenesis, as the direct interaction between these neurons and blood vessels governs a specific phase of vascular network formation in 3D, distinct from the 2D growth of ECs in the SL or the formation of the middle and deep vascular plexuses. Most of the previously well-characterized mutants mainly show defects in the formation of the intra-retinal vasculature plexuses, which are regulated by the precise production of HIF1a/2a-induced VEGF signaling for vascular patterning [30,31,61] (Table 1). In contrast, the RGC-specific *Piezo2* knockouts reveal a distinct phase of structural patterning of penetrating vessels in the third dimension, impacting the overall organization of the vascular lattice. Interestingly, as mentioned above, a previous study reported that certain undefined RGCs fine-tune local VEGF via the expression of VEGFR2 receptors, regulating the density of penetrating vessels from the superficial layer [37]. However, the mechanisms involving perivascular neurons suggest unique cell–cell interactions characterized by direct contact between neurons and vessels. It is important to note that, unlike the VEGFR2-driven regulation, the *Piezo2*-dependent mechanism guides the spatial orientation of penetrating vessels rather than their density. The detailed molecular mechanisms downstream of *Piezo2* in perivascular neurons are still unknown. Previous studies on *Piezo1* have shown that it is involved in mechano-sensitive channel-dependent calcium influx, leading to remodeling of the actin cytoskeleton at contact points and alterations to the extracellular matrix [62]. To further uncover the potential molecular and cellular mechanisms, it is critical to characterize the specific RGC and vascular components involved in these direct interactions. On the neuronal side, perivascular RGCs can be labeled genetically using transgenics. In contrast, on the vascular side, candidates include the D-tip cells [39], a population of ECs that may serve as substrates for these interactions (Figure 2a). The protocols established for single-cell RNA sequencing of the D-tip cells may help identify the molecular components involved in the selective RGC-EC interactions. They could further be expanded using spatial transcriptomics. These analyses will be particularly revealing when comparing EC subtypes and the molecular changes induced by neuron-specific *Piezo2* deletion during the critical stage of penetrating vessel sprouting around P8 (Figure 3e and f).

It is essential to highlight research on non-neuronal cells in the retinal structure that have direct cell-to-cell contacts affecting the organization of the vascular structure. While studies have emphasized the crucial role of astrocytes in regulating the vasculature of the superficial layer, recent findings suggest that the middle and deep vascular plexuses are mainly surrounded and influenced by Müller glia. Grimes et al. used high-resolution serial electron microscopy-based reconstruction to demonstrate that Müller glia covers middle layer vessels in a tessellating manner, leaving some gaps for pericyte contacts. Through pharmacological experiments, they also showed that calcium signaling from Müller glia is important for regulating vasodilation [63]. Müller glia plays a significant role in the developmental phase of ML and DL angiogenesis. Understanding whether and how they drive this process through direct contact can provide deeper insights into the regulation of vascular organization in 3D.

Another important aspect of non-neuronal cells in neurovascular regulation is the role of pericytes. Recent studies discovered that tube-like structures serving as physical connections between neighboring pericytes are crucial for vascular communication [13]. Using genetic labeling and high-resolution live imaging, Alarcon-Martinez et al. demonstrated that these structures, termed as inter-pericyte tunneling nanotubes (IP-TNTs), regulate microcircuit-level blood flow by wrapping around capillaries in the mouse retina. Calcium influx in one pericyte induces vessel dilation, which is transmitted to a connected distal pericyte via these nanotubes, resulting in a concurrent constriction response [13]. Given the intricate subcellular contacts and communication involved, it is tempting to hypothesize that intercellular protrusions known as tunneling nanotubes (IP-TNTs) may also participate in the development of retinal vasculature in 3D to regulate vascular physiology. Notably, while this retinal study reported that IP-TNTs form between two *bona fide* pericytes located on separate capillary systems, further investigation is warranted to determine whether these regional and structural differences within the CNS bear any critical functional relevance.

### Outlook with new technologies

Recent technological advancements have significantly advanced our understanding of retinal vascular development, shifting the field from traditional 2D analyses to more complex 3D investigations over the past decade. First, the advancement of single-cell transcriptomics has enabled the high-throughput identification of the molecular profiles of all retinal neuronal or glial types [12,64]; as well as cellular components of vasculature [39]. Second, by integrating insights from neuronal and vascular cells, spatial transcriptomics now allows us to map putative cell–cell interactions between these distinct cell types associated with their molecular identities [65,66]. However, significant challenges remain in characterizing vascular ECs using single-cell RNA-sequencing and spatial transcriptomic methods, as these cells constitute only 1 % of the total retinal cell population in the retina [67]. Newer approaches involving enhanced cell isolation techniques, more sensitive sequencing methods, and better computational tools may improve the characterization of these low-abundance cells. Third, recent studies have employed AAV techniques with optimized AAV sero-types for vascular delivery, as well as for labeling of CNS neurons, including those in the retina [15,68–70]. These trans-vascular labeling methods can be combined with genetic perturbations using *in vivo* CRISPR-based editing, significantly increasing the efficiency of screening for mutations that cause retinal neurovascular defects. Fourth, researchers can decipher the details of neuron–vessel interactions by integrating highresolution imaging techniques, particularly serial electron microscopy reconstructions that can yield physiological insights into neurovascular dynamics [74]. The recent findings have given us valuable insights into the vascularization process and the arrangement of blood vessels mediated by the nervous system. Nonetheless, our understanding of the mechanisms governing this neurovascular crosstalk is still in its early stages. Many ophthalmological diseases, such as retinopathy of pre-maturity and diabetic retinopathy, involve disruptions in retinal vascular organization. Therefore, further exploration of the mechanisms underlying neurovascular interactions is critical for elucidating the root causes of these conditions.

## Figures and Tables

**Table 1:** Phenotypes of mouse mutants with vascular patterning or structural deficits.

Neural Cell Types Involved	Molecular Players	Technical Approaches	Findings	References
Astrocytes and Müller glia	VEGF	Genetic perturbations (mouse Cre lines), primary cell cultures	• Astrocyte-derived VEGF is important for guiding endothelial tip cells during developmental angiogenesis. • Müller glia-derived VEGF is dispensable in developmental angiogenesis but is important in hypoxic pathological conditions.	[29,33,35,71]
RGCs, amacrine cells, and horizontal cells	VEGF	Genetic perturbations (mouse Cre lines, siRNA knockdown), Drug-induced perturbations (Diphtheria Toxin)	• VEGF from amacrine cells and horizontal cells is essential for the development and maintenance of intraretinal vasculature. • VEGFR2, expressed on retinal ganglion cells (RGCs), regulates VEGF levels and directs vascular growth. • Hypoxic retinas accumulate succinate, sensed by RGCs via the GPR91 receptor. This triggers the secretion of VEGF and angiogenic factors to promote angiogenesis.	[11,34,35,37]
Müller glia	TGFβ/TGFBR2 signaling	Genetic perturbations (mouse Cre lines), single-cell RNA sequencing	• Müller glia secrete TGFβ to promote vascular development. • TGFBR2 is specifically enriched in a subtype of ECs called the D-tip cells, which guide deep-layer vascularization.	[38,39]
Müller glia	Norrin/Fzd4/Lrp5	Genetic perturbations, Immunoaffinity purification assays, and Microarray	• Müller glia secretes Norrin during retinal angiogenesis. • Norrin binds to the Frizzled receptor (*Fzd4*), co-receptor *Lrp5*, and the co-activator *Tspan12*, which are expressed on the ECs during retinal development.	[26,42,44,72]
RGCs	Sema3E/PlexinD1	Gene perturbations (Antibody/Drug based inhibition/activation of molecules, RNA Interference), *in vitro* cultures, ligand-receptor binding assay	• PlexinD1 is expressed in ECs, and its expression is promoted by VEGF signaling. • RGCs express Sema3E, which binds to PlexinD1 in ECs to promote vessel growth and branching. • The Sema3E – PlexinD1 signaling pathway negatively regulates the VEGF-induced Notch signaling pathway, which governs the cell fate determination between tip cells and stalk cells.	[48,49]
Horizontal cells, Bipolar cells, and amacrine cells	Slit 1/2 Robo 1/2/4	Genetic perturbation (Cre lines, siRNA), Protein quantification	• *Slit1* is expressed in horizontal cells P5 onwards. *Slit2* is expressed in most bipolar neurons and some amacrine cells. • *Slit2* signals through *Robo1* and *Robo2* on endothelial cells to promote vascular development.	[73]
Starburst amacrine cells (SACs)	Cholinergic signaling	Genetic perturbations, cell ablation, Dye perfusion assay	• Cholinergic neuronal activity of SACs promotes angiogenesis. • The cholinergic neuronal input modulates VEGF signaling to regulate vessel growth and independently regulates the Wnt/Norrin signaling for BRB function.	[57]
Retinal neurons (photoreceptors and bipolar cells))	Glutamatergic signaling	Genetic perturbations, singlecell RNA sequencing, Dye perfusion assay	• Glutamatergic neuronal activity promotes retinal deep plexus angiogenesis and blood-retinal barrier (BRB) maturation. • Glutamate stimulates Norrin expression in Müller glia and interneurons, with glutamate transporters being essential for this glutamate-driven Norrin expression.	[58]
Dopaminergic RGCs	Dopamine signaling	Genetic perturbations (Cre lines, AAV-based viral injections)	• Early postnatal dopamine produced by RGCs, and not by dopaminergic amacrine cells, controls vessel growth. • Tyrosine hydroxylase production in RGCs aligns with vessel emergence. • RGC-derived dopamine promotes vessel growth by modulating Notch signaling.	[59]
Perivascular Fam19a4/Nts-RGCs	Piezo2-mediated direct neurovascular contacts	Genetic ablation, conditional mutants, transvascular viral labeling, Electrophysiological measurements	• Fam19a4/Nts-RGCs directly contact blood vessels with perisomatic endfeet. • Ablation of Fam19a4/Nts-RGCs disrupts the perpendicular penetrating vessel growth. • Fam19a4/Nts-RGCs utilize neuronal Piezo2 to guide 3D vascular lattice organization. • Disorganized 3D vascular lattice impairs capillary perfusion and visual function.	[60]

## Data Availability

No data was used for the research described in the article.
